# A dataset of storm surge reconstructions in the Western North Pacific using CNN

**DOI:** 10.1038/s41597-024-03249-5

**Published:** 2024-04-22

**Authors:** Wen Dang, Jianlong Feng, Delei Li, Mengzhen Fan, Liang Zhao

**Affiliations:** 1 Key Laboratory of Marine Resource Chemistry and Food Technology (TUST), Tianjin University of Science and Technology, Tianjin, 300457 China; 2 Key Laboratory of Ocean Observation and Forecasting, Key Laboratory of Ocean Circulation and Waves, Institute of Oceanology, Chinese Academy of Sciences, Qingdao, China

**Keywords:** Physical oceanography, Hydrology, Physical oceanography

## Abstract

The relatively short duration of available tide gauge records poses challenges for conducting comprehensive statistical analyses of storm surges in the Western North Pacific. To address this issue, we employ a convolutional neural network model to reconstruct the maximum daily storm surge at 160 tide gauges from 1900 to 2010 in the Western North Pacific. The reconstructed dataset serves multiple purposes. Firstly, it facilitates the identification of regions where notable changes in the storm surges have occurred in the past. Additionally, the dataset enables long-term analyses of the storm surge climate, offering insights into historical patterns and variations. Furthermore, it provides a solid foundation for conducting robust extreme value analyses. To ensure accessibility, the data are publicly available through a repository, allowing for easy access and utilization by the broader scientific community and the general public. Overall, our research contributes to the field of oceanography by providing a dataset that aids in understanding the historical storm surge dynamics in the Western North Pacific region.

## Background & Summary

The Western North Pacific (WNP) Ocean’s warm pool is the origin of about 60% of all tropical cyclones (TCs; also known as typhoons), bringing the WNP a significant risk of damaging storm surges^1–5^. Notable instances of storm surges that have resulted in significant loss of life and property include Super Typhoon Haiyan in 2013, Typhoon Tiantu in 2013, Typhoon Rammasun in 2014, and Typhoon In-Fa in 2021. Within the 1-meter coastal flood plain, there are approximately 73 million people residing in the WNP^6^. Therefore, a better understanding of storm surges is imperative to facilitate a more accurate assessment of flood risk.

In recent years, there has been a lot of attention to investigating how climate change affects the storm surges in the WNP^7,8^. Some studies have examined changes in storm surge extremes with large-scale indices such as the PDO and ENSO using tide gauge data^2,9,10^. Using model simulation, Oey and Chou^11^ demonstrate a marked increase in the intensity of typhoon surges, as well as a discernible trend of poleward-shifting occurring after the 1980s. This shift can be attributed to the weakening of the steering flow within the tropics, which is a consequence of global warming. However, due to the data limitations, most studies have only been conducted since 1970s and 1980s, making it difficult to gain a comprehensive understanding of the variability and changes in storm surges across time and space in the WNP.

To address the scarcity of data records, considerable efforts have been made in storm surge data reconstruction. These efforts encompass observations, dynamic numerical techniques, and data-driven methodologies, each demonstrating their own advantages and limitations^12^. The SURGEDAT database serves as a global repository for storm surge events, documenting the location and magnitude of tropical surge occurrences worldwide since 1880. Now it has archived the location and height of more than 700 tropical surge events around the world^13,14^. Muis *et al*.^15^ have successfully generated the Global Tide and Surge Reanalysis dataset, covering the period from 1979 to 2014. This achievement was accomplished by employing hydrodynamic storm surge models, which were driven by the ERA-interim data. In a subsequent study, Muis *et al*.^16^ produced a notable global dataset of extreme sea levels for the period from 1979 to 2017. Muis *et al*.^17^ produced the global sea level change time series for 1950–2050, which was derived from reanalysis and high-resolution CMIP6 climate projections, including tides, storm surges, and sea level rise. These advancements were facilitated by employing the third generation Global Tide and Surge Model. Despite their achievements, the implementation of dynamic numerical techniques presents operational complexities and substantial expenses. The setup process is intricate, and its execution requires significant computational resources and time. Additionally, these techniques heavily rely on long-term, high-resolution atmospheric reanalysis data. Consequently, these factors limit the generation of long-term hindcasts of storm surges. Conversely, data-driven methods offer notable advantages compared to numerical models, as they require relatively low resolution for meteorological data and minimal computational resources. Cid *et al*.^18^ utilized multiple linear regression methods to construct a global storm surge database from 1871 to 2010 on a global scale. After that, Cid *et al*.^19^ utilized a similar approach to reconstruct daily maximum storm surges from 1866 to 2012 within Southeast Asia. Moreover, Tadesse and Wahl^20^ employed machine learning methods to simulate the maximum daily fluctuations along the global coastline. However, the accuracy of these studies varies spatially, for example the reconstruction correlation coefficients for storm surge data in the Western North Pacific were relatively low compared to the coasts of Europe and North America. This observation is particularly prominent in southern China, certain regions of Japan, and Malaysia.

In contrast to traditional reconstruction techniques, neural network methods can automatically learn and fit parameters without relying on oversimplified assumptions in the data reconstruction process^21^. In particular, neural networks are effective in representing non-linear relationships between different variables^22^. In recent years, data-driven neural network approaches have shown great potential in the study of data reconstruction in geoscience^23–26^. For example, by using an improved neural network model, Wang *et al*.^24^ estimated the ocean subsurface thermal structure from 0–2000 m from multisource sea surface data in the western Pacific Ocean. Smith *et al*.^26^ proposed a convolutional neural network architecture for the reconstruction of vertical profiles of temperature and salinity between 2005 and 2020 in Atlantic Ocean. Additionally, neural networks are also used in storm surge forecasting and warning in recent years, including the back-propagation neural networks, artificial neural network, and convolutional neural network^27–29^. The results indicate that compared to traditional statistical or machine learning methods, neural networks provide more accurate storm surge forecasts. However, most studies focused on storm surge forecasting using neural network, with fewer studies on storm surge data reconstruction.

**Fig. 1:**
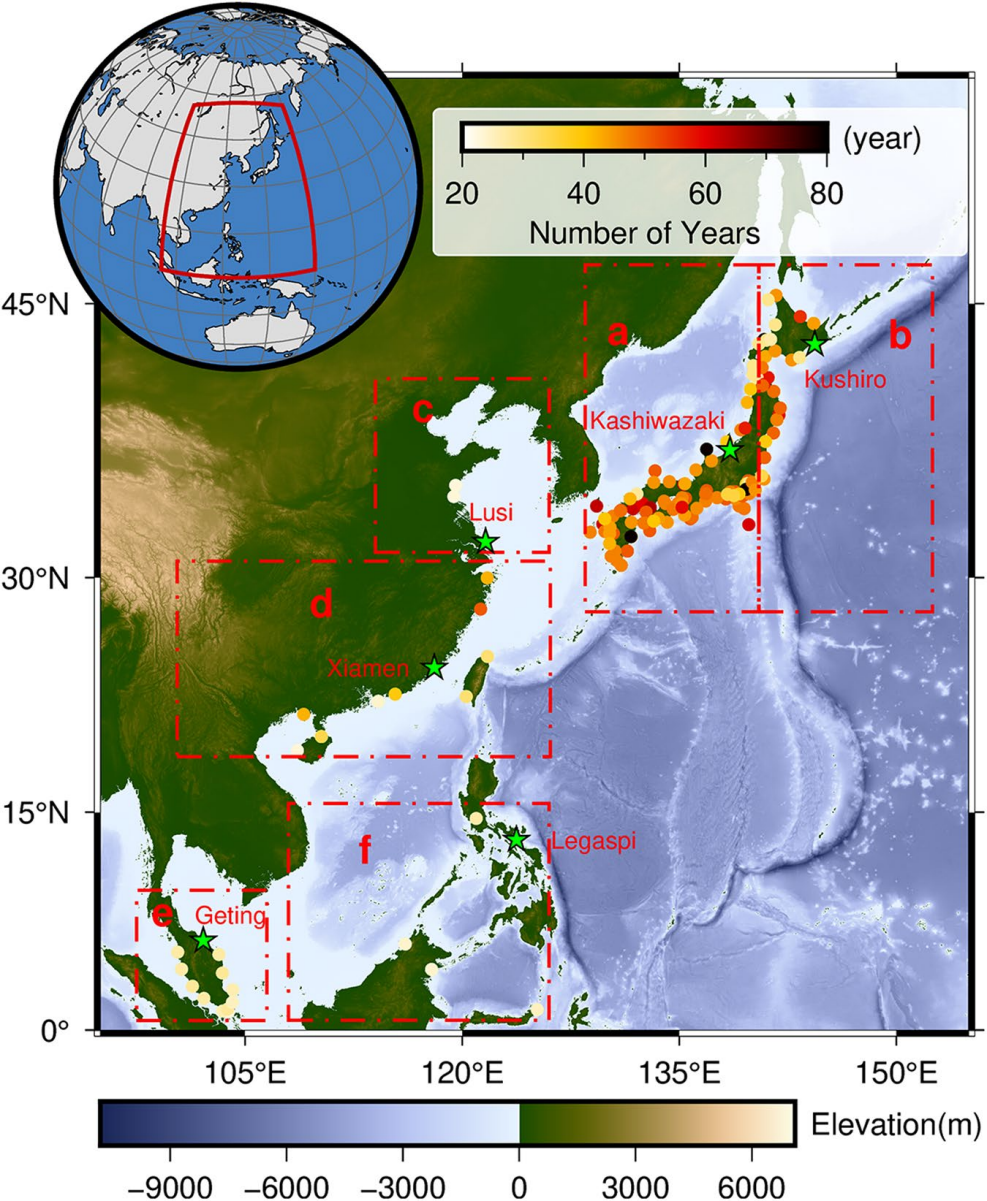
Distribution map and regional division of all stations in Western North Pacific. The color indicates the length (years) of the tide gauge records. Green pentagrams represent validation sites of tide gauges in the Technical Validation section to assess model performance in details.

**Fig. 2:**
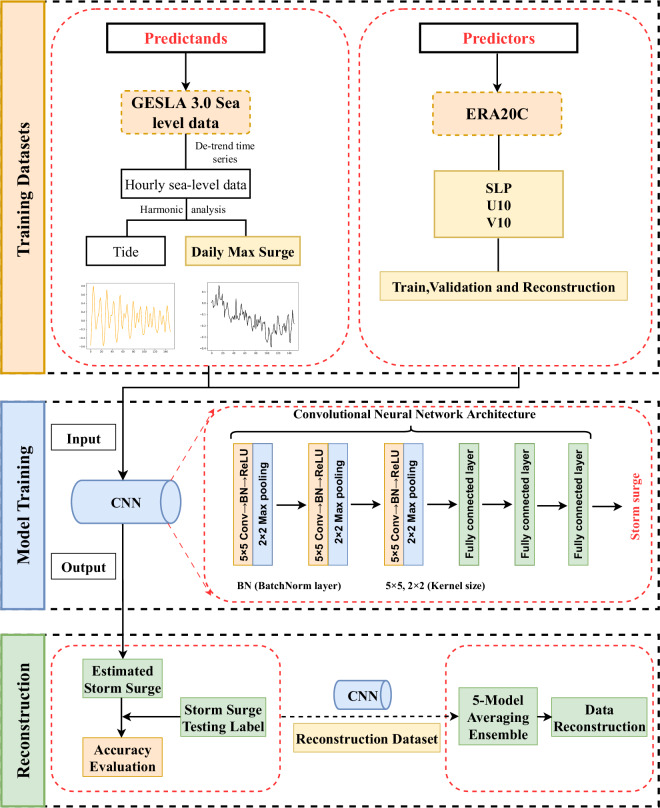
Schematic diagram of the overall workflow. These steps include training datasets, model training, and reconstruction.

In this paper, by using the convolutional neural network (CNN) we presented a reconstructed dataset of the daily maximum storm surges of 1900–2010 in the WNP (Fig. 1). The overall workflow for the database development is depicted in Fig. 2. The long-term surge reconstruction can be used to do more accurate storm surge assessment as well as to better understand trends and longer-term fluctuations in the storm surge climate.

## Methods

The goal of this work is to reconstruct daily maximum storm surge values in WNP (100°E-145°E, 0°N-55°N) from 1900 to 2010. This is accomplished by training a functional relationship between the daily maximum storm surge values (predictand) and the surrounding atmospheric conditions (predictor) using the CNN model. Our methodology consists of five principal stages, delineated as follows. The initial stage involves data acquisition, wherein we gathered the ERA20C and GESLA-3 datasets for the Western North Pacific region. The subsequent stage is dedicated to data preprocessing, wherein the primary task is to execute a series of data manipulations to align with the input requirements of the CNN model. The third stage encompasses the training of the CNN model, utilizing the processed datasets at each respective site. The fourth stage pertains to the validation of results, wherein the performance metrics of the validation set are evaluated. The final stage is the reconstruction of storm surge data.

### Data sources

The predictors were obtained from ECMWF’s atmospheric reanalysis of the 20th century (ERA-20C) provided by the European Centre for Medium-Range Weather Forecasts (ECMWF) (https://www.ecmwf.int/)^30^. The ERA-20C has a longitudinal resolution of 1.125°, a latitudinal resolution of 1.121°, and a temporal resolution of 3 hours. The dataset covers the period from 1900 to 2010.

Daily maximum storm surge data were extracted from The Global Extreme Sea Level Analysis (GESLA-3) datasets^31^. Tide gauges located between 0°N and 55°N latitude and 100°E and 145°E longitude were specifically selected in the analysis. In order to ensure the accuracy of the CNN, tide gauges with fewer than 20 years of recorded data prior to 2010 were excluded from the dataset. The spatial distribution of the tide gauges and available years of the data over 20 years are shown in Fig. 1. The final dataset comprised 160 tide gauges, and the mean data record length is approximately 42 years. To calculate the storm surge at each location, the predicted tide was subtracted from the actual water level using the Python UTide package^32^.

### Predictor pre-processing

Three variables, including 10 metre zonal wind (U10) and 10 metre meridional wind (V10) speeds, and mean sea-level pressure (SLP), were used as the predictors in the CNN model. We also tested the inclusion of variables such as precipitation, and temperature in the model, however, the additional variables cannot improve the performance of the model. The predictors (U10, V10, and SLP) are extracted from within a 10° × 10° box around each tide gauge. To allow the model to learn more features, we interpolated the predictor variables to a resolution fields of 0.25° × 0.25° using the linear interpolation method. Every box for the tide gauges had 40 × 40 data grid points with a time range of 1900–2010. After trying different days (one day, two days, three days and four days) of predictors corresponding to the daily maximum storm surge, we found two days to be the optimal choice considering model accuracy and computation time. In our experimental design, the predictor encompasses all available data points within a two-day period. Specifically, the ERA20C dataset post-interpolation offers a temporal resolution of three hours and a spatial resolution of 0.25°. As an example, at an individual site, we utilize a grid with dimensions of 10° by 10°, resulting in sixteen (8 per day) two-dimensional 40 × 40 arrays for a single variable over the span of two days. For the analysis of three atmospheric variables (U10, V10, and SLP), we generate three separate two-dimensional arrays, each with dimensions of 40 × 40. These are then concatenated to produce a three-dimensional array with dimensions of 48 × 40 × 40, where each variable is represented by a distinct channel within the data structure. Predictand for one day corresponds to predictor variables for the previous two days (one day including predictand). For illustrative purposes, if the daily maximum storm surge on the 2nd of January, 2000, is selected as the predictand, then the predictors from both the 1st and 2nd of January, 2000, are utilized as inputs to the model. We standardized the preprocessing of the data for each variable so that they were on the same scale. We then combined the data from the three variables into a matrix that meet the needs of the model. The data-driven models can then be trained and reconstructed using the predictor matrix. The dataset of predictors corresponding to the data with observed daily maximum storm surge was set as the training datasets from 1900 to 2010. On the contrary, the dataset of predictors corresponding to the data without observed daily maximum storm surge was set as the reconstruction datasets.

### Predictand pre-processing

The daily maximum storm surge (named as the predictand in the CNN) is standardized before being trained in the model. After training, the output of the model is standardized inversely to get the daily maximum storm surge. Experiments show that the method helps in the fitting of the model.

### Convolutional neural network model

CNN utilize convolutional layers to efficiently extract spatial feature information from input data, necessitating fewer parameters relative to fully connected neural networks^21,33,34^. Furthermore, an Artificial Neural Network (ANN) was also employed for the reconstruction of datasets related to the maximum daily storm surge. A comprehensive comparison underscores the ANN’s significantly lesser performance in comparison to the CNN for this specific application. The architecture of the convolutional neural network (CNN) model utilized in this study is depicted in Fig. 2. Our experimental iterations are conducted in three primary dimensions: variable selection, hyperparameter optimization, and model architecture design. In the context of variable selection, we have incorporated additional variables, such as temperature and precipitation, to assess their impact on the model’s performance. The hyperparameter tuning process primarily entails the selection of learning rates, loss functions, and optimizers. The model architecture is meticulously crafted, taking into consideration the number of layers, the quantity of channels within each layer, and the choice of activation functions. Through numerous experimental iterations, a design featuring three convolutional layers followed by corresponding pooling layers, as well as three fully connected layers, was chosen as the model architecture^35,36^ (Fig. 2). Rectified Linear Units (ReLU) were employed as the activation function, while Stochastic Gradient Descent (SGD) served as the optimizer. Similar to most fitting problems, we used Mean Squared Error Loss (MSELoss) as the loss function. To mitigate overfitting and prevent the occurrence of exploding or vanishing gradients, Batch Normalization (BN) was applied after the convolutional layers.

### Model training, validation, and reconstruction

The pre-processed predictor was utilized to train the CNN model and validate its performance. To improve the accuracy of the reconstructed storm surge data, a strategy involving a 5-model averaging ensemble was implemented^37^. Initially, all data sets were divided into five equal partitions, with one partition assigned as the holdout set. This process was repeated five times, ensuring that a different partition served as the holdout set during each repetition. Subsequently, the reconstructed datasets from the five models were averaged together, resulting in the final reconstructed dataset. By comparing, we found that this ensemble approach effectively reduced the impact of randomness and significantly contributed to the enhanced accuracy of the reconstructed storm surge data.

Using the 5-model averaging ensemble method, five models were trained on the training set, resulting in five predictions on the validation set. The predictions were then averaged to obtain the final reconstructed dataset. Furthermore, individual training was performed at each station as a final step, and the results were evaluated using various statistical analyses.

The validation set division was employed to evaluate the accuracy of the reconstructed dataset at all tide gauge locations. To assess the accuracy, metrics such as Pearson’s correlation coefficient, Root Mean Square Error (RMSE) and Mean Absolute Error (MAE) were utilized. These metrics provided a quantitative measure of the correlation and the difference between the reconstructed and observed data, respectively.

$$Pearson{\prime} s\;Correlation({r}_{xy})=\frac{{\sum }_{i=1}^{n}({x}_{i}-\bar{\mathrm{x}})({y}_{i}-\bar{\mathrm{y}})}{\sqrt{{\sum }_{i=1}^{n}{({x}_{i}-\bar{\mathrm{x}})}^{2}}\sqrt{{\sum }_{i=1}^{n}{({y}_{i}-\bar{\mathrm{y}})}^{2}}}$$

$$RMSE=\sqrt{\frac{{\sum }_{i=1}^{n}{\left({y}_{i}-{x}_{i}\right)}^{2}}{n}}$$

$$MAE=\frac{{\sum }_{i=1}^{n}\left|{y}_{i}-{x}_{i}\right|}{n}$$

Where x and y stand for the values of the observed and modelled surges, respectively, and $\bar{\mathrm{x}}$ and $\bar{\mathrm{y}}$ for their respective means. We also use Relative RMSE (RRMSE) as another evaluation metric to assess the accuracy of the reconstructed data. The RRMSE was obtained by normalizing RMSE by the maximum surge variability observed at a specific tide gauge.

Despite the statistics in terms of Pearson’s correlation, RMSE, and RRMSE for all tide gauges, we also selected six sites (Kushiro, Kashiwazaki, Lusi, Xiamen, Geting, and Legaspi) randomly in different regions and divide the dataset into validation and training sets. The validation datasets consisted of the first five years of the original dataset, while the remaining data were allocated as the training datasets. We specifically analysed the performance of the model on the validation set and on all datasets. And the validation is done in terms of extreme values. Furthermore, the model’s performance was tested using several storm surge events caused by typhoons.

Once the training and validation phases are complete, surge reconstruction will be performed using the predictor matrix of the reconstructed dataset.

## Data Records

This dataset for the surge reconstruction contains README files, folders that contains the surge reconstructions for tide gauges in WNP, and metadata folders with the model validation results (both for daily maximum surges and extreme surges above the 95^th^ percentile). For individual tide gauges, daily maximum surge time surges are stored in comma-separated value (.csv) format. The dataset is available at Figshare^38^.

## Technical Validation

**Fig. 3:**
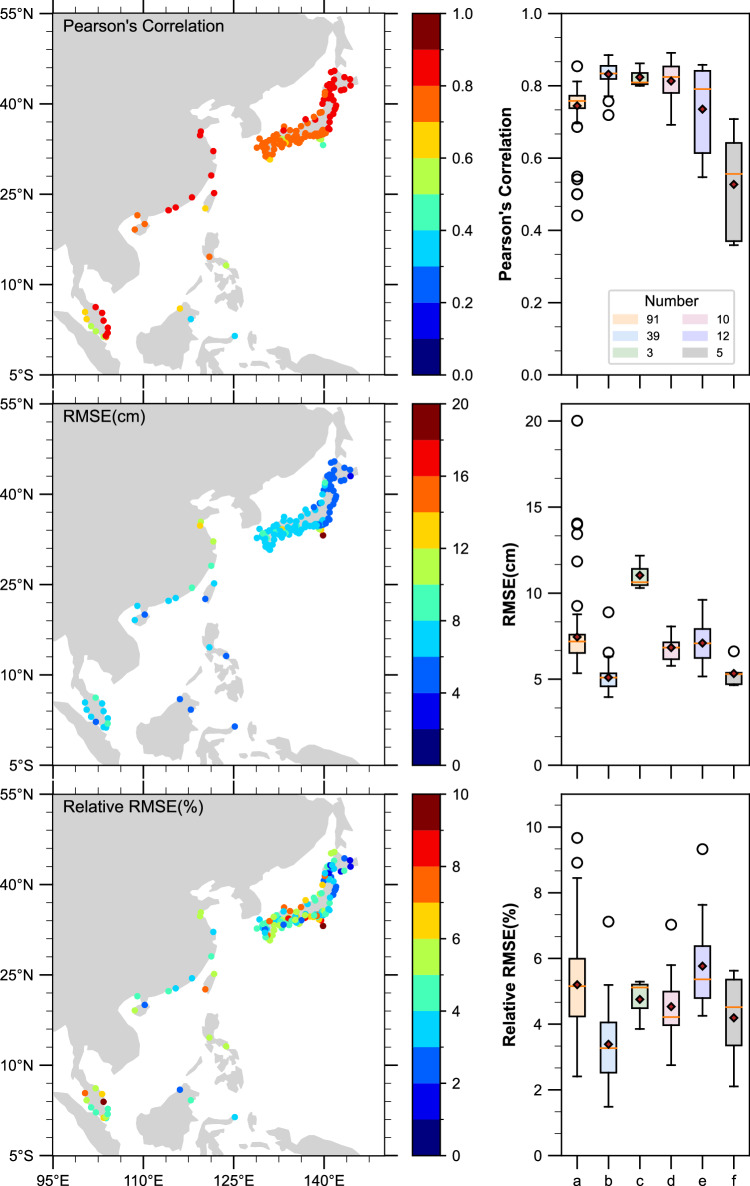
Model validation results. Validation of surge reconstructions in all datasets in terms of Pearson’s correlation (top), RMSE (middle), and RRMSE (bottom). Right panes show the variability of the metrics in different partitions.

We reconstructed the daily maximum storm surge level from 1900 to 2010, employing the aforementioned methods. To evaluate the accuracy of our surge reconstructions, we compared the simulation results with corresponding observed daily maximum surge values (Fig. 3). The left panes of Fig. 3 depict the variability in performance metrics for the surge reconstructions. On the right panes, we present the spatial distribution (Fig. 1) of the validation results for the surge reconstructions. Our analysis revealed promising results, with an average correlation coefficient of 0.76 (std = 0.09) and root mean square error (RMSE) of 6.8 cm (std = 1.99). Most of the sites exhibited correlation coefficients above 0.7 and 0.8, accounting for 89% and 34% of the total, respectively. The highest correlation coefficient obtained was 0.89. Notably, regions b, c, and d demonstrated correlation coefficients above 0.8 on average, outperforming other regions. These regions primarily encompass areas in eastern Japan and China. Overall, higher latitudes yielded superior results compared to lower latitudes. These findings align with those reported by Tadesse and Wahl^20^. Additionally, Tadesse *et al*.^12^ supported these conclusions by demonstrating that wind speed and SLP exhibit more pronounced variations at higher latitudes, and some reanalyses better capture these patterns. Additionally, the surge reconstructions in northern Malaysia (region a) exhibited strong performance, with correlation coefficients above 0.8 in all instances. The majority of tide gauges yielded RMSE values below 8 cm, amounting to 88% of the total gauges. Region b displayed the lowest RMSE, with an average of 5 cm. Region g also exhibited relatively low RMSE values, predominantly below 6 cm. Furthermore, our analysis revealed an average relative RMSE (RRMSE) of 4.7%, with 89% of the total falling below 7%. The results indicate the overall accuracy and reliability of our surge reconstructions, suggesting their suitability for oceanographic studies.

**Fig. 4:**
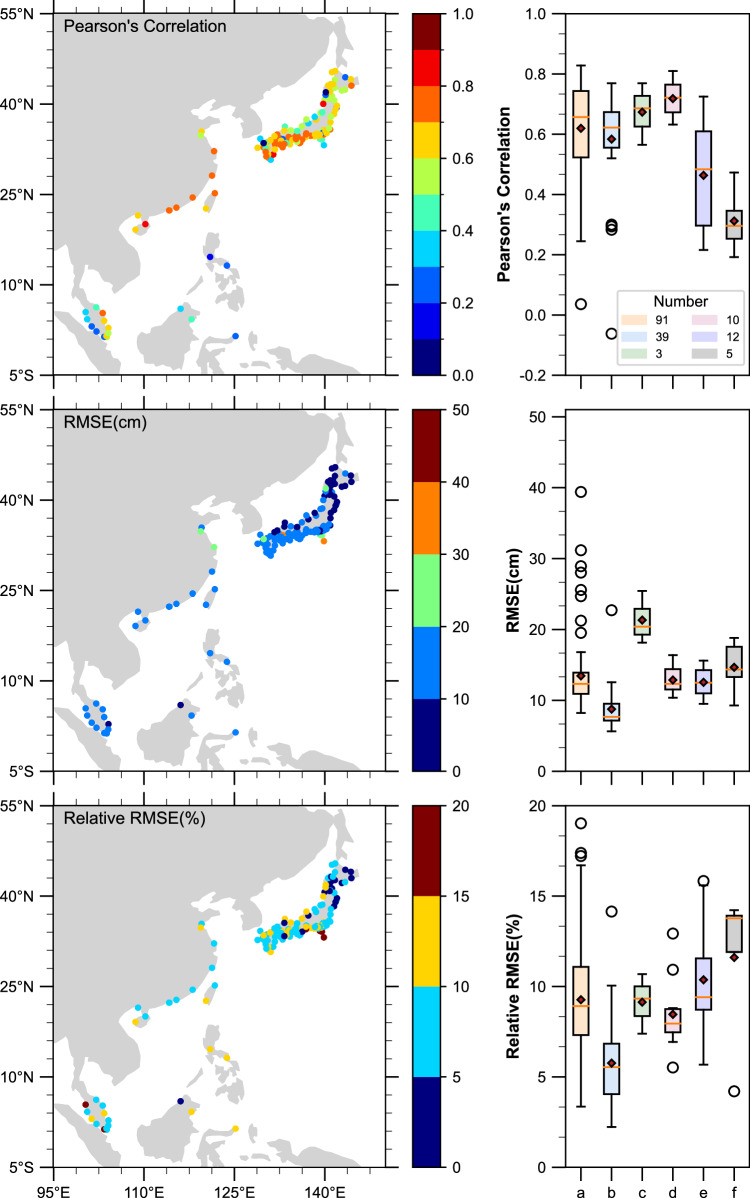
Model validation results for extreme events. Extreme values validation of surge reconstructions in all datasets in terms of Pearson’s correlation (top), RMSE (middle), and RRMSE (bottom) for extreme surge events (above the 95% percentile). Right panes show the variability of the metrics in different partitions.

We conducted further verification of the accuracy of storm surge extremes across all stations. To assess the performance of our simulations, we compared the simulated values with observed surges exceeding the 95th percentile threshold at each tide gauge (Fig. 4). Our analysis revealed an average correlation coefficient of 0.6 (std = 0.16) and a root mean square error (RMSE) of 12.4 cm (std = 4.78). The highest correlation coefficient achieved was 0.83. Notably, 30% of tide gauges exhibited correlation coefficients exceeding 0.7, primarily in western Japan and along the coastal regions of China. Region d demonstrated the highest correlation coefficient, with an average value of 0.75. In terms of the RMSE, 28% of all sites had values below 10 cm. The region b, located in eastern Japan, which yielded the most favourable validation results with mean RMSE values below 10 cm. Additionally, the average relative RMSE (RRMSE) was found to be 8.5%, with 73% of the total falling below 10%. These findings highlight the overall accuracy and reliability of our simulation results, especially in relation to storm surge extremes.

We also conducted a comparison of our simulation results with the data-driven models developed by Tadesse and Wahl^20^. They performed reconstructions of global storm surge data and assessed the accuracy of their reconstructions from 1980 to 2010. To ensure a fair comparison, we focused on sites within the WNP (Western North Pacific) region and excluded sites with less than 30 years of storm surge data. For the daily maximum storm surges, we obtained correlation coefficients, root mean square error (RMSE), and relative RMSE (RRMSE) values of 0.77, 6.9 cm, and 4.7%, respectively. These results have shown improvement relative to those data generated by Tadesse and Wahl^20^, who achieved correlation coefficients of 0.45, an RMSE of 7.76 cm, and an RRMSE of 7.4%. Furthermore, we assessed the extreme values of storm surges and obtained correlation coefficients, RMSE, and RRMSE values of 0.62, 12.4 cm, and 8.3%, respectively. In contrast, Tadesse and Wahl^20^ reported a correlation coefficient of 0.37, an RMSE of 17 cm, and an RRMSE of 15.6% for extreme values. Our simulation shows a higher correlation coefficient and lower error metrics, indicating the advantage of neural network methods in storm surge reconstruction compared to the traditional reconstruction techniques.

**Fig. 5:**
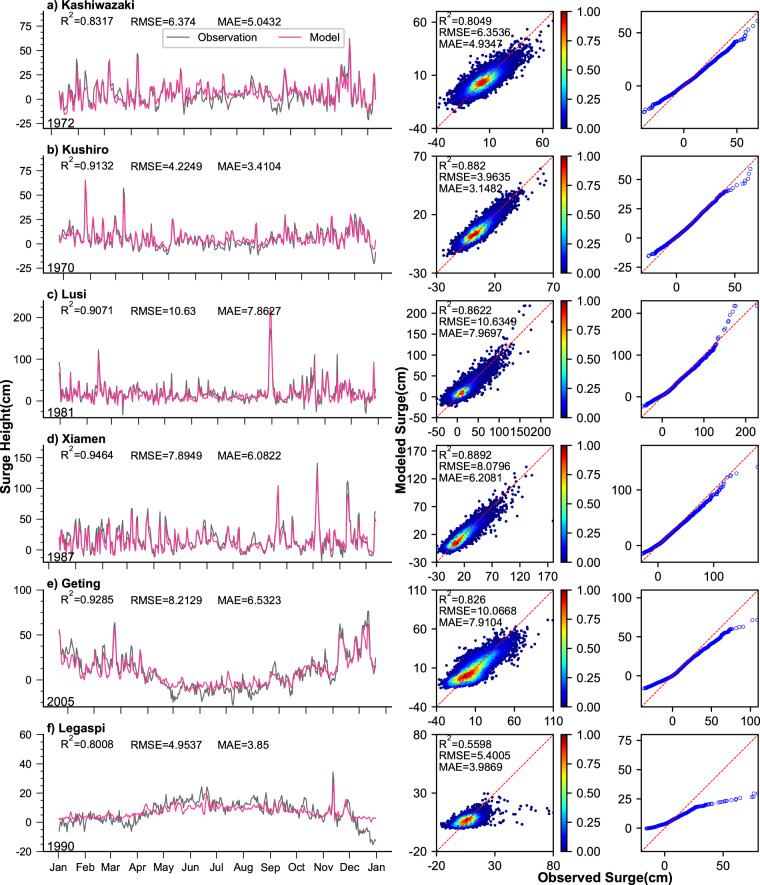
Model validation results in all data sets at six validation locations. Daily maximum surge time series comparison (left), scatter plots (middle), and quantile-quantile plots (right) for observed and modelled daily maximum surge for six tide gauge locations (marked in green pentagram in Fig. 1).

Additional validation was performed by comparing observed and modelled daily maximum surge time series, scatter plots, and quantile-quantile (Q-Q) plots at six validation locations scattered across different regions (Fig. 1). The results for these stations are presented in Fig. 5. The correlation coefficients varied between 0.56 at Legaspi (Philippines) and 0.89 at Xiamen (China), while the root mean square error (RMSE) ranged from 3.9 cm at Kushiro (Japan) to 10.6 cm at Lusi (China). The MAE ranged from 3.1 cm at Kushiro (Japan) to 8 cm at Lusi (China). It is noteworthy that the highest correlation coefficient of 0.95 was achieved at Xiamen (China) in the time series plots, with an associated RMSE of 7.9 cm, and an MAE of 6 cm. The model performed well in predicting extreme values in this case (Fig. 5, left). However, most sites demonstrated a slight underestimation of extreme value predictions, except for Lusi (China) (Fig. 5, right).

**Fig. 6:**
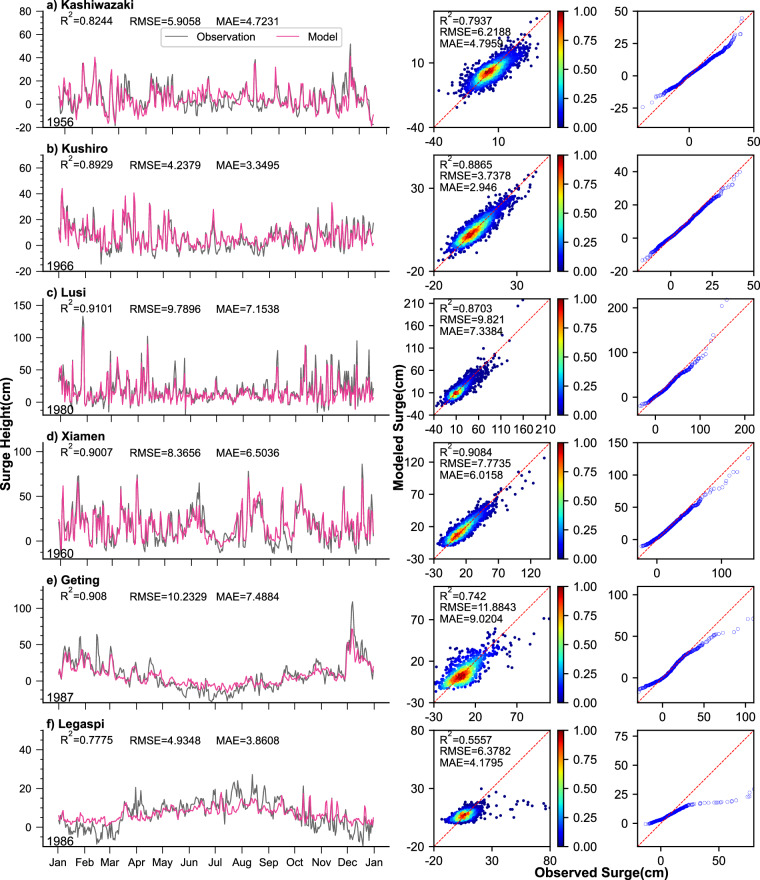
Model validation results in the validation set (the first five years) at six validation locations. Daily maximum surge time series comparison (left), scatter plots (middle), and quantile-quantile plots (right) for observed and modelled daily maximum surge for six tide gauge locations (marked in green pentagram in Fig. 1).

The model’s performance on the validation datasets is notable, exhibiting consistency across all datasets (Fig. 6). The time series plots depict the validation set for one random year (3 before the satellite, 3 after the satellite). The correlation coefficients varied between 0.56 (Legaspi (Philippines)) and 0.91 (Xiamen (China)), while root mean square error (RMSE) ranged from 3.7 cm (Kushiro (Japan)) to 11.9 cm (Geting (Malaysia)) (Fig. 6, middle). The MAE ranged from 2.9 cm at Kushiro (Japan) to 9 cm at Geting (Malaysia). Notably, the time series comparison displayed the highest correlation coefficient of 0.91 at Lusi (China), indicating very good performances in daily maximum storm surge reconstruction. The metrics for both the entire dataset and the validation sets closely aligned, underscoring the model’s strong generalization capabilities. Overall, the CNN model demonstrates a good fit for storm surge data reconstruction, capturing the general patterns and variations. The high correlation coefficients and relatively low RMSE values indicate a good fit between the observed and modelled surge data. However, it should be acknowledged that the model slightly underestimates extreme values, as indicated by the Q-Q plots, particularly at Geting (region e) and Legaspi (region f). This underestimation of extremes may be attributed to the limited availability of extreme value samples in the training data, resulting in incomplete assimilation of information pertaining to such storm surge events. Consequently, future model training should consider further refinement and inclusion of a broader spectrum of extreme events.

**Fig. 7:**
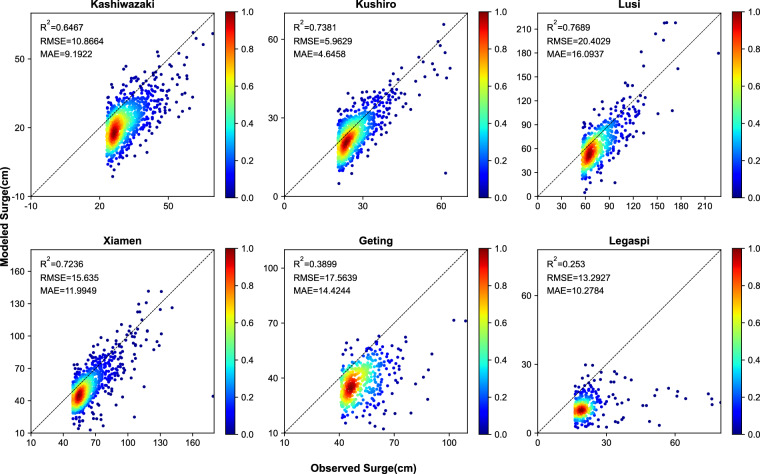
Validation of Model Performance for extreme values at six validation locations (surge values above the 95^th^ percentile threshold in all datasets).

**Fig. 8:**
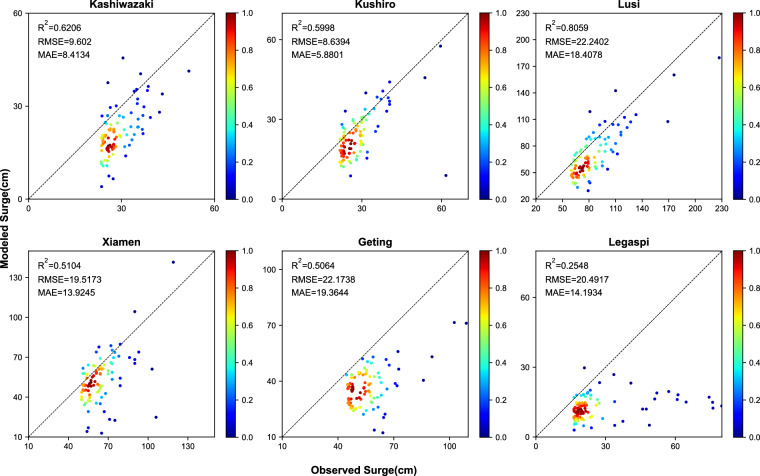
Validation of Model performance for extreme values at six validation locations (surge values above the 95^th^ percentile threshold in the validation set (the first five years)).

Additionally, we validate the reconstructions of the surge focusing on extreme events. Figure 7 illustrates the differences between actual and simulated values above the 95th percentile threshold for six tide gauges. The number of extreme value data points was determined by selecting the maximum 5% of quantities from the validation set data. Four of the sites exhibited correlation coefficients above 0.5, reaching up to 0.77 at Lusi, while the RMSE varied from 6 cm at Kushiro to 20.4 cm at Lusi. The MAE ranged from 4.6 cm (Kushiro) to 16 cm at Lusi. Similarly, 95 thresholds were calculated on all datasets (Fig. 8). Five of the six validation sites had correlation coefficients above 0.5 (but Legaspi), with a maximum correlation coefficient of 0.81 (Lusi) and RMSE ranging from 8.6 cm (Kushiro) to 22.2 cm (Lusi). The MAE ranged from 5.9 cm (Kushiro) to 19.4 cm at (Geting). The model demonstrates a good fit for extreme storm surges; however, the simulated values tend to be slightly lower compared to the overall validation set data.

**Fig. 9:**
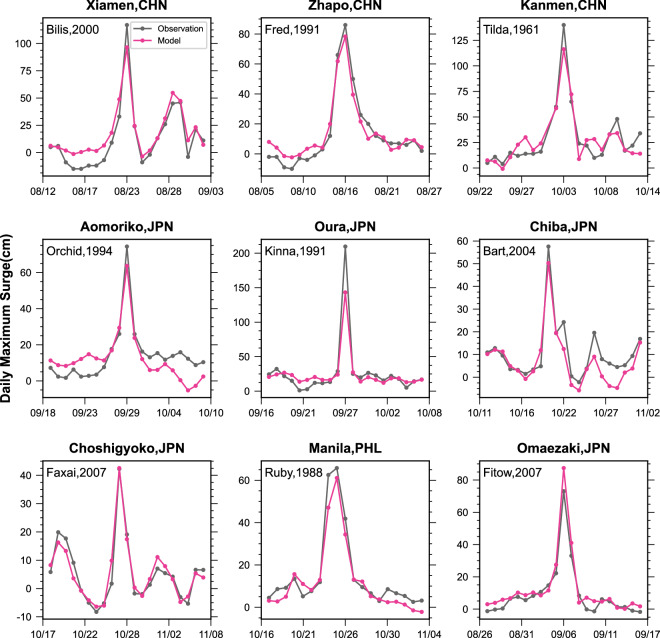
Validation of surge reconstruction for individual storm events.

Besides that, model consequences for specific extreme events are also validated. The maximum daily change in storm surge as a typhoon passes over the tidal stations was shown in Fig. 9. The average relative deviation of the typhoon-induced maximum storm surge is 14.4%. The model captures the change in the daily maximum storm surge as the typhoon passes over the station, especially at Zhapo (the typhoon Fred, 1991), and at Omaezaki (the typhoon Fitow, 2007). However, there are individual station peaks that are underestimated, such as Qura (Japan). One possible reason is that the sample size of such very large storm surge events is too small for the model to learn.

In summary, this study leverages a convolutional neural network (CNN) model to emulate the daily maximum storm surges in the Western North Pacific from 1900 to 2010. The empirical analysis demonstrates that CNN is efficacious in the reconstruction of storm surge data. The reconstructed dataset yielded encouraging results across all monitored stations, with an average correlation coefficient of 0.76, a Root Mean Square Error (RMSE) of 6.8 cm, and a relative RMSE of 4.7%. Notably, a significant fraction of sites, constituting 34% of the total, exhibited correlations exceeding 0.8. Our findings not only corroborate the accuracy of our storm surge reconstructions in the WNP region but also demonstrate significant improvements over existing data-driven models. The reconstructed dataset facilitates an in-depth analysis of storm surge patterns, their variability, and the assessment of extreme values in the Western North Pacific. The findings of this research hold the potential to inform the scientific community, emergency management professionals, and coastal infrastructure designers about the coastal flood risks specific to the Western North Pacific region.

The reconstruction of storm surge data at individual sites is predicated upon the atmospheric conditions specific to each location, suggesting that the methodologies delineated in this study are adaptable for application across various basins. It is anticipated that the model presented herein can be extrapolated for broader utilization across diverse geographic regions. Future research may endeavor to refine the predictors at a more granular level, thus enabling the convolutional neural network (CNN) to reconstruct storm surge data with greater precision. For instance, the fifth-generation European Centre for Medium-Range Weather Forecasts (ECMWF) atmospheric reanalysis of the global climate (ERA5) dataset, which boasts a finer spatial resolution, spans from January 1940 to the present. The ERA5 dataset could potentially be employed to downscale low-resolution, long-term datasets through a neural network, thereby obtaining a higher resolution driving field for more accurate modeling.

However, in contrast to dynamic numerical methods, data-driven approaches such as neural networks do not inherently resolve the intricacies of spatial distribution. Presently, our capacity to reconstruct datasets is confined to tide stations with existing data. Unlike traditional methodologies, the neural network paradigm necessitates independent network training for each station to enhance accuracy, which may potentially increase the workload. In contrast to SURGEDAT^13^, our daily maximum storm surge datasets don’t include the tidal results, so we can’t get the changes of extreme sea level and the high water marks.

## Usage Notes

The complete reconstruction data for each tide gauge in the WNP can be downloaded by users^38^.

The reconstructed dataset can be used to conduct research on the multi-scale changes of storm surge, and can also be used as an observational reference to explore changes of storm due to anthropogenic climate change. In addition, it can provide more accurate standards for the design of ocean engineering. However, users should also be aware of certain disadvantages. For example, studies have demonstrated that the centennial reanalysis products from the early 20^th^ and 19^th^ centuries are of lower quality^39^. Users should interpret the results of the full centennial surge reconstructions with caution. Additionally, the accuracy of reconstructed data at some sites performs poorly in the tropical regions. We plan to further update the storm surge dataset reconstruction, including longer and more accurate storm surge reconstruction datasets.

## Data Availability

The python code that was used to train the model to generate the dataset descripted above can be found at Figshare^38^. Further questions can be directed towards Jianlong Feng (fjl181988@tust.edu.cn).
